# NK cell exhaustion: bad news for chronic disease?

**DOI:** 10.18632/oncotarget.5490

**Published:** 2015-08-22

**Authors:** Jamie L. Schafer, Michaela C. Müller-Trutwin, R. Keith Reeves

**Affiliations:** Center for Virology and Vaccine Research, Beth Israel Deaconess Medical Center, Boston, MA, USA

**Keywords:** natural killer, HIV, cancer, lymphocyte exhaustion, Immunology and Microbiology Section, Immune response, Immunity

Natural killer (NK) cells are generally considered as the effector arm of nonspecific innate immunity, providing rapid recognition and lysis of virus-infected and tumor cells, but can also play significant regulatory roles in pregnancy, cooperate with T and B cell responses, and even exhibit independent adaptive memory. However, much like other lymphocyte populations, recent burgeoning evidence suggests that in chronic conditions NK cells can become functionally exhausted. T cell exhaustion is generally induced by constant exposure to persistent antigens resulting in a loss of effector functions and an overall decline in resistance to disease, and NK cell exhaustion appears to share many similar features. One of the best-studied examples of NK cell dysregulation is in HIV infection and in SIV-infected nonhuman primate models. Indeed, recent work from our laboratory found that NK cells in lymph nodes of SIV-infected rhesus macaques are continuously activated, likely by low-level ongoing virus replication [1]. This continuous activation resulted in in situ differentiation followed by functional anergy, as indicated by a failure to lyse target cells and upregulated Tim-3 expression. These data helped clarify empirical observations in both HIV and SIV infections of impaired cytotoxic function, altered cytokine production and impaired antibody-dependent cell-mediated cytotoxicity (ADCC) [2]. Indirect effects of HIV/SIV include the consistent bombardment of NK cells with target cells expressing dysregulated HLA and systemic cytokines, resulting in net exhaustion. Interestingly, similar indicators of upregulated Tim-3, PD-1 and lytic failure have all been found in CMV, Hepatitis C and Hepatitis B infections. Together, these data indicate NK cell exhaustion in chronic stages of viral disease may be directly related to a loss of control of virus replication and/or reactivation of latent virus reservoirs.

In addition to chronic viral infections, NK cell exhaustion has also been observed in several human cancers and animal tumor models. Much like NK cells in SIV infection and previous descriptions of exhausted T cells, common features of NK cells in cancer and cancer models are high expression of inhibitory receptors including Tim-3, downregulated activating receptors, and decreased expression of transcription factors, EOMES and T-bet [3–5]. Furthermore, mouse models have demonstrated that NK cell dysfunction due to inefficient phosphorylation in activation signaling pathways arises only against MHC-deficient tumors, not against tumors that maintain MHC expression [6]. Indeed, just repeated exposure of NK cells in the presence of tumors can reduce cytotoxic activity [3]. While NK cell exposure to tumors can reduce cytotoxic activity, excess proliferation also contributes to NK cell dysfunction, as even homeostatic proliferation may induce an exhausted state in the absence of a tumor. Exhaustion of NK cells is also highly specific, as NK cell defects are far more severe in tumor-associated NK cells than in peripheral NK cells from the same individual or animal [3, 5, 6]. The clinical relevance of decreased NK cell function is also evident in disease progression, a prime example being breast cancer where NK cells with reduced function and high expression of inhibitory receptors are associated with more aggressive and invasive tumors [5].

NK cell exhaustion in chronic diseases appears to have many conserved features, including upregulation of inhibitory receptors and excess proliferation due to virus or cytokine-induced activation. Furthermore, NK cell recognition is highly dependent on ‘self’ and ‘missing-self’ discrimination through MHC binding, and repeated NK cell interaction with target cells where MHC is lacking, either by viral or oncogenic etiology, is a prominent cause of exhaustion. Although in its infancy, therapeutic restoration of NK cell numbers and functions are being developed. Preliminary success reverting NK cell exhaustion by therapeutic overexpression of EOMES [3], blocking of Tim-3 [4], or cytokine stimulation by IL-12, IL-18 or an IL-2 derivatives [6] have improved NK cell anti-tumor responses. Exogenous IL-12, IL-15 and/or IFN-α have also proven beneficial in reactivating NK cell function in nonhuman primate models [7]. Additionally, nonpathogenic hosts of SIV infection do not appear to exhibit evidence of NK cell exhaustion despite high virus replication [8], and these models could inform future strategies to augment NK cell function. In summary, these data highlight the concept that functional exhaustion of NK cells is associated with loss of control of chronic viral infections and inadequate elimination of neoplastic cells, but current strategies for augmentation of impaired NK cell function are underdeveloped and should be considered for future therapies.
